# Supplementary material for: Impact of the COVID-19 Pandemic on the Incidence and Characteristics of Patients with Psychiatric Illnesses Visiting Emergency Departments in Korea
Source: J Clin Med. 2022 Jan 18;11(3):488. doi: 10.3390/jcm11030488 (PMC8836502; doi:10.3390/jcm11030488)
Supplement: Supplementary file 1 [file jcm-11-00488-s001.zip › jcm-1520939-supplementary.pdf]

Table S1. Distribution of principal diagnostic codes for all ED visits with psychiatric illnesses

Diagnosis group	Total		Before-COVID		During-COVID	
	N	%	N	%	N	%
Total	161,801		88,520		73,281	
Main diagnosis (ICD-10)						
Neuropsychology (F)	74,419	46.0	41,706	47.1	32,713	44.6
Infection and organ specific disease (endocrine, brain, circulatory, respiratory, gastrointestinal) (A, B, E, G, I, J, K)	37,601	23.2	20,287	22.9	17,314	23.6
Injury (S, T)	19,243	11.9	10,052	11.4	9,191	12.5
Urology, OBGY, neonatal disease (N, O, P, Q)	4,442	2.7	2,274	2.6	2,168	3.0
Eye, dermatology, musculoskeletal (H, L, M)	3,210	2.0	1,835	2.1	1,375	1.9
Cancer (C, D)	2,898	1.8	1,565	1.8	1,333	1.8
Nonspecific abnormality and other specific code (R, U, V, Z)	19,988	11.4	10,801	11.4	9,187	11.5

(A to Z): Diagnosis group of ICD-10.

COVID-19, coronavirus disease 2019; ED, emergency department; ICD-10, International Classification of Diseases, 10th edition
